# Preparation of ZIF-8 and Its Application in Determination of Pyridoxine and Pyridoxal in Ginkgo Seeds by Ultra-Performance Liquid Chromatography

**DOI:** 10.3390/foods11142014

**Published:** 2022-07-07

**Authors:** Yuan Gao, Mengjia Xu, Zhe Zheng, Yiqun Wan, Shihang Wu, Chang Li

**Affiliations:** 1 College of Chemistry, Nanchang University, Nanchang 330047, China; gaoyuan12460@163.com (Y.G.); xumj1122@163.com (M.X.); wanyiqun@ncu.edu.cn (Y.W.); 2 State Key Laboratory of Food Science and Technology, China-Canada Joint Laboratory of Food Science and Technology (Nanchang), Key Laboratory of Bioactive Polysaccharides of Jiangxi Province, Nanchang University, Nanchang 330047, China; zhengzhe1121@163.com; 3 Affiliated School, Nanchang University, Nanchang 330047, China; shihang_wu@163.com

**Keywords:** ginkgo seeds, pyridoxine, pyridoxal, ZIF-8, UPLC

## Abstract

A new rapid and accurate method was developed for simultaneous determination of pyridoxine and pyridoxal in ginkgo seeds, using ultra-performance liquid chromatography (UPLC) equipped with a fluorescence detector. Diluted hydrochloric acid solution was used as the extracting solvent. For the pretreatment of extracts, a zeolitic imidazolate framework material (ZIF-8) was prepared and characterized. An ODS-BP column (4.6 mm × 250 mm × 5 μm) was used for separation. The conditions of sample extraction, cleaning and separation were optimized. The linear correlation coefficient (R^2^) of the analyte was better than 0.9999, indicating good linearity. The limits of detection (LODs) of pyridoxal and pyridoxine were 0.0065 mg/kg and 0.0057 mg/kg, respectively, and limits of quantitation (LOQs) were 0.022 mg/kg and 0.019 mg/kg, respectively. The recovery of the two substances ranged from 86.2% to 110.4%, and the relative standard deviation (n = 6) was less than 7.5%. The method was applied to determine the contents of pyridoxine and pyridoxal in actual ginkgo seed samples with satisfactory results.

## 1. Introduction

*Ginkgo biloba* L. is one of the oldest relict plants in the world [1]. It originates from China and is called “living fossil” [1]. The ginkgo tree, which grows slowly but lives for an extremely long time, is found mainly in China, Japan and South Korea. Ginkgo seeds are rich with nutritional and medicinal value [2], and have been used as foods and medicine for thousands of years in East Asia [3].

However, overconsumption of ginkgo seeds could lead to poisoning. In 1985, Wada et al. [4] isolated 4-*O*-methylpyridoxine (MPN) from ginkgo seeds for the first time and speculated that MPN was the main toxic substance causing symptoms of ginkgo poisoning. The main symptoms of ginkgo poisoning are convulsions and loss of consciousness. In 1988, Wada et al. [5] adopted multi-step separation combined with animal experiments, based on the poisoning symptoms caused by the isolates as the judgment basis, and finally, successfully isolated and obtained the toxic components, identified as MPN. In 2000, Scott et al. [6] used the Soxhlet extraction method, combined with HPLC, LC-MS and LC-MS-MS, for the first time, to extract and detect 4-*O*-methylpyridoxe-5-glucoside (MPN-5-glucoside, MPNG) from ginkgo seeds. Currently, MPN and MPNG are collectively known as ginkgo toxin and are considered to be the main toxic substances in ginkgo seeds. A case reported that high MPN concentrations were observed in both the serum and cerebrospinal fluid from a 2-year-old girl who ingested 20–30 ginkgo seeds [7]. Studies have shown that ginkgo toxin content in ginkgo seeds is affected by various factors, such as producing area, growing period and variety. Gong et al. [8] investigated the content changes in MPN, MPNG and vitamin B6 compounds in ginkgo seeds at different growth stages and found that the total content of vitamin B6 compounds in ginkgo seeds decreased with the increase in growth period, while the total content of ginkgo toxin increased continuously. This suggests that vitamin B6 compounds are related to MPN, and vitamin B6 could be a precursor of MPN biosynthesis. Vitamin B6 is a general term for a class of pyridine compounds that can be converted into each other, including pyridoxamine, pyridoxine, pyridoxal and their phosphoric acid compounds at the 5’ position. Both MPN and MPNG are derivatives of vitamin B6 and their structures are similar. Yoshimura et al. [9] established an HPLC method for simultaneous determination of MPN, MPNG and other vitamin B6 analogues in ginkgo seeds. However, the determination of pyridoxine and pyridoxal in ginkgo seeds could be interfered by MPN, MPNG and other analogues [10]. In some cases, pyridoxal and pyridoxine need to be measured separately [11]. The interference effects, including matrix effect, could be an obstacle to the accurate determination of pyridoxine and pyridoxal.

Zeolitic imidazolate framework materials (ZIFs), such as metal organic framework materials (MOFs), not only have the advantages of periodic network structure, high specific surface area, and adjustable pore size, but also high stability in the molecular sieve and they have shown good application prospects in the field of organic compound adsorption in recent years [12,13,14]. ZIF-8 is a well-known ZIFs family member with molecular sieving properties, exhibits a sod topology comprising 1.16 nm cages connected through six-membered windows, and 0.34 nm in size [15,16]. ZIF-8 has been applied in sample pretreatment before HPLC and LC-MS/MS determination [17,18,19]. However, there are no reports on pretreatment of extracts of ginkgo seeds with ZIF-8.

In this work, a UPLC method was established for determination of pyridoxine and pyridoxal in ginkgo seeds from different origins. ZIF-8 was prepared for pretreatment of samples. This work could provide a new method for the determination of pyridoxine and pyridoxal in ginkgo seeds.

## 2. Materials and Methods

### 2.1. Chemicals and Reagents

Methanol (HPLC grade, ≥99.9%) was purchased from Thermo Fisher Technologies Co., Ltd. Ethanol (chromatographic pure, 99.9%) (Waltham, MA, USA), formic acid (≥98%), zinc nitrate hexahydrate (chromatographic pure, 99.998%) and hydrochloric acid (analytically pure, 36–38%) were purchased from Xilong Scientific Co., Ltd. China (Shantou, China). 2-methylimidazole (≥99.0%) was purchased from Dibai Biotechnology Co., Ltd., Shanghai, China. Pyridoxal and pyridoxine (HPLC grade, ≥99%) were purchased from Yuanye Biotechnology Co., LTD., Shanghai, China. Pyridoxal and pyridoxine were prepared in 0.1 mol/L hydrochloric acid to form a standard reserve solution with a concentration of 1000 mg/L and stored at 4 °C. A series of mixed standard working solutions were prepared according to experimental requirements.

### 2.2. Preparation of ZIF-8

ZIF-8 was synthesized according to the previous study [15] with a minor modification. Briefly, 2.38 g 2-methylimidazole and 2.63 g Zn(NO_3_) ·6H_2_O were dissolved in 66.4 mL methanol and stirred for 20 min at room temperature. The mixture was aged for 24 h. After centrifugation, the white solid obtained was washed with methanol 3 times and dried at 80 °C.

### 2.3. Characterization of ZIF-8

The Thermo Scientific Nicolet 6700 FT-IR spectrometer was used to determine the IR spectra of the samples in a range of 400–4000 cm^−1^ based on KBr pellets. Powder X-ray diffraction (XRD) patterns were recorded at room temperature on a Bruker D8 Advance diffractometer in transmission geometry using Cu Kα radiation (λ = 1.54059 Å) at 40 kV and 40 mA and scanning rate was 2 min^−1^. The specific surface area and pore size distribution of the samples were measured by N_2_ sorption-desorption experiment at 77 K using the static volumetric method and 3H-2000 specific surface area tester. Particle size distributions in the ZIF-8 were determined with a Malvern Zetasizer Nano ZS90 (Malvern Instruments Ltd., GB). Field emission scanning electron microscopy (SEM) was used to scan and image the morphology of samples at different multiples.

### 2.4. Sample Pretreatment

The ginkgo seeds were dried in a lyophilizer at −50 °C for 12 h. The dried seeds were crushed and passed through an 80-mesh sieve. Next, 0.5 g ginkgo seeds powder was accurately weighed and placed into a 50 mL centrifuge tube. Then, 15 mL hydrochloric acid solution with a concentration of 0.05 mol/L was added and mixed evenly by shaking. Ultrasonic-aid extraction was performed at room temperature for 15 min, and centrifugation was performed at 10,000 r/min for 10 min. The supernatant was taken and 10 mg of solid adsorbent ZIF-8 material was added. The mixture was stirred for 10 min, then stood for 5 min, and centrifuged at 10,000 r/min for 10 min. Further, 1 mL of the supernatant was filtered by 0.22 μm aqueous microporous membrane before the test.

### 2.5. UPLC Condition

Ultra-performance liquid chromatography (UPLC) was performed using an Agilent 1290 ultra-performance liquid chromatograph (Agilent, Santa Clara, CA, USA) equipped with high-pressure binary pumps and a fluorescence detector (G1321B). A SinoChrom ODS-BP column (4.6 mm × 250 mm × 5 μm) was used for separation. The determinations were carried out on the column at 30 °C. The excitation and emission wavelengths were set at 280 and 400 nm, respectively. The chromatograms were recorded and the peak areas were calculated using ChemStation software (Agilent Technologies Inc., Palo Alto, CA, USA). The solvent system was a gradient of methanol (A) and 0.3% formic acid solution (B). The gradient was as follows: 0 min: 95% B; 8.0 min: 83% B; 8.1 min: 10% B; 10 min: 10% B; and 10.1 min: 95% B. The total run duration was 15.0 min. The flow rate was 1.0 mL/min and the injection volume was 10 µL.

### 2.6. Statistical Analysis

The experiment was conducted in triplicates. The related data were statistically analyzed using SPSS statistical software with ANOVA and Dunken’s tests to signify significant variations among means (*p* < 0.05).

## 3. Results and Discussion

### 3.1. Characterization of ZIF-8

**Figure 1:**
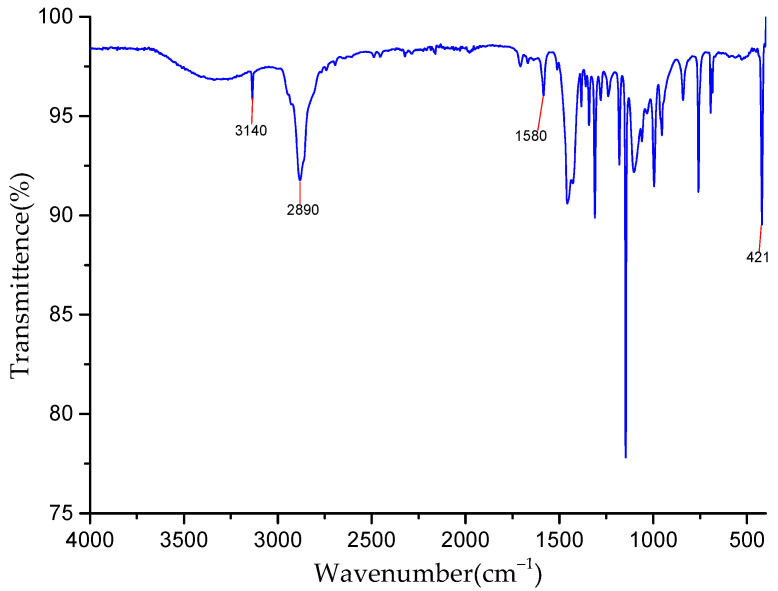
FT−IR spectra of ZIF-8.

Figure 1 shows that the peaks at 3140 cm^−1^ and 2890 cm^−1^ belong to the stretching vibration peaks of the C-H bond in methyl and imidazole rings, respectively. The stretching vibration peak of the C=N bond on the imidazole ring appears at 1580 cm^−1^, and the stretching vibration peak of Zn-N appears at 421 cm^−1^. At 2600 cm^−1^, N-H peaks of 2-methylimidazole are not found, which indicates that 2-methylimidazole was completely deprotonated in the synthetic system, and pure phase ZIF-8 was obtained.

**Figure 2:**
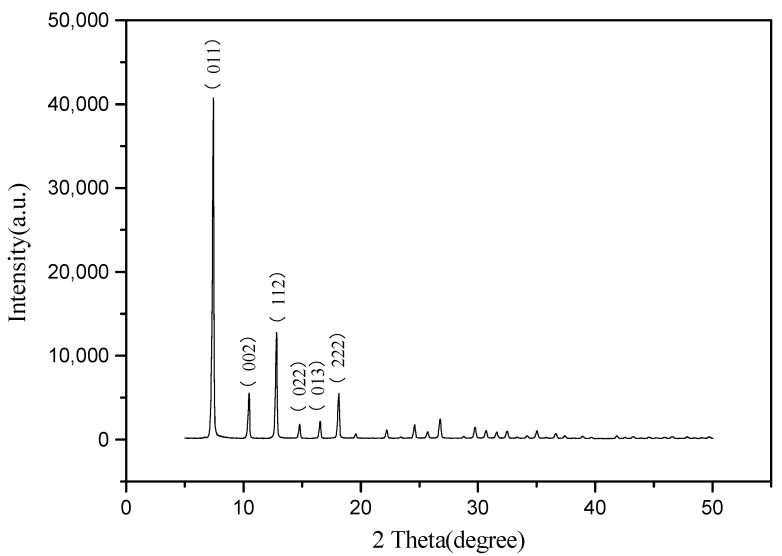
XRD pattern of ZIF-8.

It can be seen from Figure 2 that the characteristic diffraction peaks of ZIF-8 at (011), (002), (112), (022), (013) and (222) of the synthesized samples in the present study are consistent with those reported in the literature, which indicates that the synthesized samples are pure-phase ZIF-8 with high crystallinity.

The specific surface area and pore size distribution of the sample were measured by a N_2_ adsorption-desorption experiment at 77 K using the static volumetric method and 3H-2000 specific surface area tester. The specific surface area of ZIF-8 is 1458.166 m^2^/g, and the pore volume is 0.696 cm^3^/g. According to the pore size distribution diagram, the pore size of ZIF-8 is distributed around 29.7 nm, and the average pore size is 1.531 nm.

**Figure 3:**
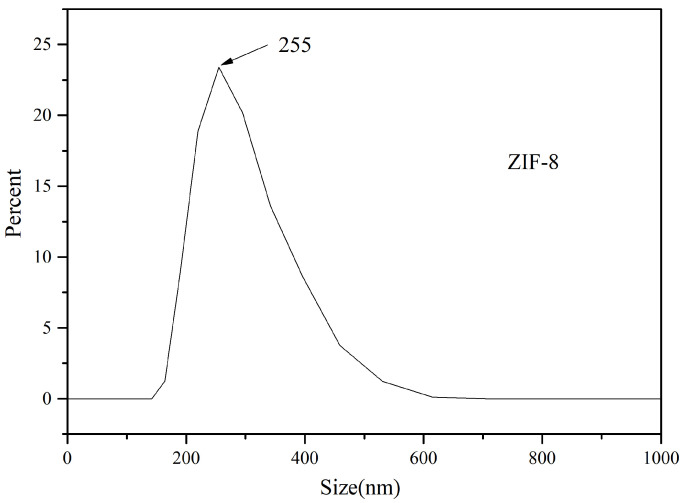
Particle size distribution of ZIF-8.

As can be seen from the cumulative distribution in Figure 3, ZIF-8 particles are evenly distributed and concentrated in a range of 0–500 nm when the optimal condition reaction is adopted, and no large particles appear. ZIF-8 particles are mono-dispersed particles, indicating that 2-methylimidazole reduces the surface energy, hinders the collision between nanoparticles, and prevents Oswald dissolution in the nanoparticles. ZIF-8 particle size presents a partial distribution on the whole, which is appropriate as a solid adsorbent.

**Figure 4:**
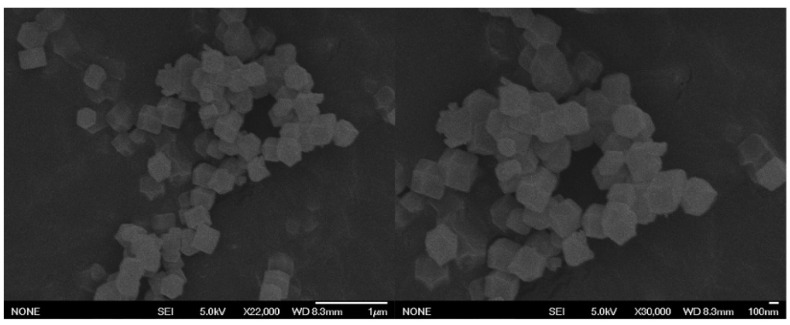
SEM characterization of ZIF-8.

Field emission scanning electron microscopy was used to scan the morphology of samples at different multiples. The particle size distribution of ZIF-8 was uniform, about 70 nm, and its morphological characteristics are shown in Figure 4.

### 3.2. Optimization of Chromatography Conditions

**Figure 5:**
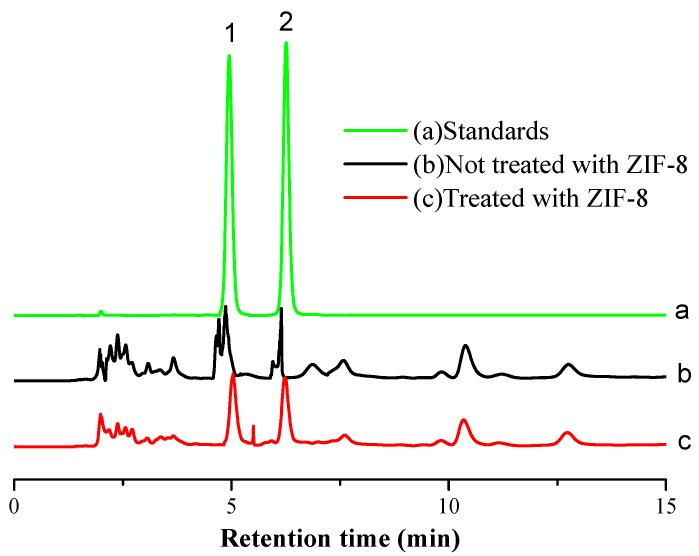
Chromatogram of standards and samples (1. Pyridoxal; 2. Pyridoxine).

In this study, the effects of mobile phase composition on the separation of pyridoxal and pyridoxine were investigated. Methanol-20 mmol/L ammonium acetate, acetonitrile-20 mmol/L ammonium acetate, acetonitrile-0.1% phosphoric acid water, methanol-0.1% phosphoric acid water, methanol-0.1% formic acid water and methanol-0.3% formic acid water were selected as mobile phases and compared for the separation. Acetonitrile and methanol were compared as mobile phase A. When methanol was used as mobile phase A, the response signal strength of pyridoxine was stronger. Meanwhile, 20 mmol/L ammonium acetate, 0.1% phosphoric acid water and 0.1% formic acid water were compared as mobile phase B. When 20 mmol/L ammonium acetate or 0.1% phosphoric acid served as mobile phase B, the baseline separation of two analytes was barely achieved and chromatographic peak tailing was observed. When 0.3% formic acid water was used as mobile phase B, a better peak shape and a better baseline separation of pyridoxine and pyridoxine were obtained. The experiment used different initial ratios of methanol to compare the gradient elution conditions. The results showed that the separation was not successful when the ratio of methanol was higher than 10%, and the separation was appropriate when it was reduced to 5%. When the ratio of methanol continued to decrease from 5%, the retention time of analytes was prolonged. Therefore, the initial ratio of methanol was selected at 5% for gradient elution in this study. The settings of gradient elution time were optimized based in many tests to obtain optimal chromatographic resolution and retention time. The results showed that pyridoxal and pyridoxine had good peak shape and could be separated well (Figure 5a), using methanol-0.3% formic acid water as mobile phase and gradient elution.

UPLC conditions: The detector was a fluorescence detector. The excitation wavelength was 280 nm; the emission wavelength was 400 nm. The separation was carried out on a C18 column. The column temperature was 30 °C. The mobile phase consisted of methanol (A) and 0.3% formic acid solution (B). The gradient program was as follows: 0 min: 95% B; 8.0 min: 83% B; 8.1 min: 10% B; 10 min: 10% B; and 10.1 min: 95% B. The total run duration was 15.0 min. The flow rate was 1.0 mL/min and the injection volume was 10 µL.

### 3.3. Optimization of the Extraction Conditions

**Figure 6:**
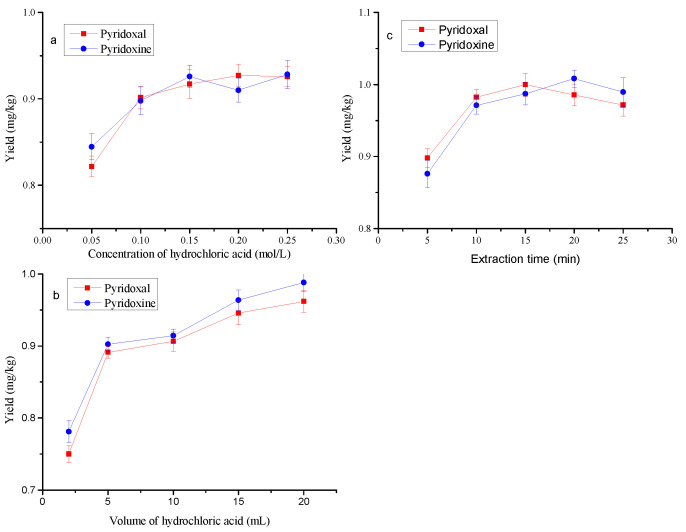
Effects of concentration and volume of hydrochloric acid and extraction time on the yields. (**a**) Extraction with 10 mL for 15 min; (**b**) extraction with 0.15 mol/L for 15 min; (**c**) hydrochloric acid concentration and volume were 0.15 mol/L and 10 mL, respectively.

The previous study showed that hydrochloric acid extraction could increase the liberation of pyridoxal and pyridoxine from the samples [20]. Therefore, hydrochloric acid solution was used as an extraction solvent in the present study. ZIF-8 absorbent was used to purify the extract before determination by UPLC. The effects of extraction solvent dosage, concentration and extraction time on the yields were investigated. The results are shown in Figure 6a–c. With the increase in hydrochloric acid concentration, the yields were on the rise; when the concentration changed from 0.10 mol/L to 0.25 mol/L, there was no significant difference in the yields among them (*p* > 0.05) (Figure 6a). Therefore, the concentration of hydrochloric acid 0.10 mol/L was suitable for the extraction. With an improvement in the volume of hydrochloric acid, higher yields were obtained, and there was no significant difference between the dosages of 15 mL and 20 mL (*p* > 0.05) (Figure 6b), which indicates that the volume of hydrochloric acid could be set at 15 mL. In addition, it can be seen from Figure 6c that the effects of extraction time on the yields were not significant for 10, 15, 20 and 25 min (*p* > 0.05). Consequently, 10 min was selected as the extraction time in this study.

### 3.4. Optimization of Purification Conditions

**Figure 7:**
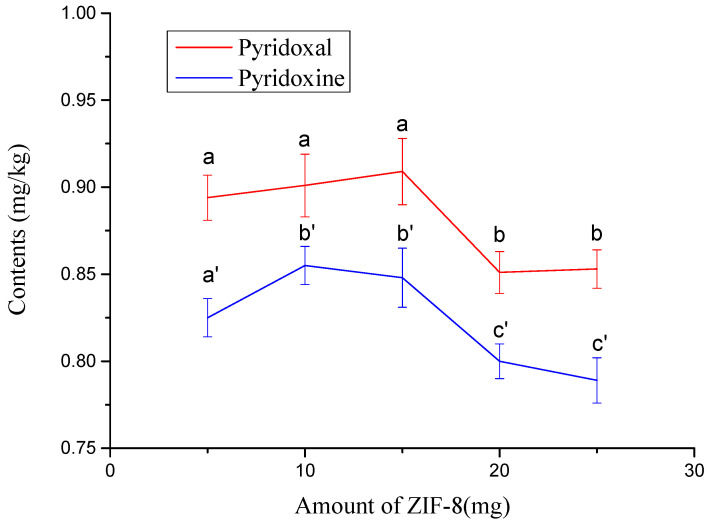
Effects of amount of ZIF-8 on measured contents of pyridoxal and pyridoxine. The values not sharing a common letter (a, b) or (a’, b’ and c’) are significantly different (*p* ≤ 0.05).

Considering the complexity of the ginkgo seed extract, some coexisting substances may interfere with the detection of target substances and shorten the service life of the chromatograph column. The effect of the ZIF-8 adsorbent on the purification of the extract was investigated. Figure 5b illustrates that a poor chromatogram was obtained as the extract was not treated with ZIF-8. As shown in Figure 5c, a cleaner extract was obtained when it was pretreated with ZIF-8. The amount effects of ZIF-8 on the measured contents of target compounds were also investigated. The results showed that the measured content of the target compound decreased significantly (*p* ≤ 0.05) when the amount of ZIF-8 was over 15 mg (Figure 7). When the amount of ZIF-8 was 10 and 15 mg, the measured contents of pyridoxal and pyridoxine had no significant differences between the two amounts (Figure 7). Therefore, 10–15 mg of ZIF-8 is suitable for pretreatment. In this study, 10 mg was chosen as the amount of ZIF-8.

### 3.5. Linearity, Limits of Detection and Quantification

Calibration curves were obtained using the mixed standard solutions to assay method linearity. A series of mixed standard working solutions with different concentrations were prepared and determined under the proposed conditions. The peak area of each compound was used for linear fitting of the concentration of the analyte to obtain the working curve by least-squares linear regression analysis. The limits of detection (LOD) and limits of quantification (LOQ) values of the investigated analytes were assessed using signal-to-noise (S/N) ratios of 3 and 10, respectively; the results are shown in Table 1. The linear correlation coefficient (R^2^) of the analyte was better than 0.9999, indicating good linearity. The LODs of pyridoxal and pyridoxine were 0.0065 mg/kg and 0.0057 mg/kg, respectively, and LOQs were 0.022 mg/kg and 0.019 mg/kg, respectively.

### 3.6. Recoveries and Precision

Ginkgo seed samples without pyridoxal and pyridoxine were used as the matrix to study recovery. Three mixed standard solutions with different concentration levels were added in samples, respectively, and the acid extraction and ZIF-8 pretreatment were followed. Each spiked level was measured in parallel six times, and the results are shown in Table 2. The recoveries of the target substances ranged from 86.2% to 110.4%, and the relative standard deviation (RSD, n = 6) was less than 7.5%, indicating that this method has good accuracy and precision and can be used for the analysis of actual samples.

### 3.7. Pyridoxine and Pyridoxal Determination in the Samples

Ginkgo seed samples from 10 different regions in China were determined by the established analytical method and the results are shown in Table 3. The contents of pyridoxal and pyridoxine in ginkgo seeds ranged from <LOD to 1.26 mg/kg and <LOD to 26.96 mg/kg, respectively. The content of pyridoxine was higher than that of pyridoxal in most samples. Pyridoxal and pyridoxine exist in the samples from Chengdu, Sichuan, and Guilin, Guangxi, with similar content level. A sample collected from Xinyang, Henan, was detected without pyridoxal and pyridoxine. The content of pyridoxal and pyridoxine in the samples varies greatly, which may be caused by different soil, climate and picking period in different regions [1,10,21]. A previous study [8] reported that the total content of vitamin B6 compounds in ginkgo seeds decreased with an increase in growth period, and pyridoxal-5′-phosphate was considered the main form of vitamin B6 compounds in the ripening period of ginkgo seeds. Therefore, this could explain why the levels of the two compounds varied widely from sample to sample, or even were not detected. Previous studies reported that MPN was converted from pyridoxal through the DXP-independent pathway or derived via the 4′-*O*-methylation of pyridoxine [22]. Based on this **finding**, it can be speculated that ginkgo seeds with low contents of vitamin B6 may exhibit more toxicity.

## 4. Conclusions

A new UPLC method using ZIF-8 for sample pretreatment was established for the analysis of pyridoxal and pyridoxine in ginkgo seeds. In this paper, solid-material ZIF-8 was prepared and characterized. ZIF-8 showed good performance in the sample purification. To the best of our knowledge, this is the first time that samples have been pretreated with ZIF-8 in the determination of pyridoxal and pyridoxine in ginkgo seeds. The role of ZIF-8 is to adsorb impurities in the sample and purify the sample to be tested. The research expands the use of ZIF-8. Optimization of the sample pretreatment and chromatographic separation conditions was also investigated. Dilute hydrochloric acid was optimal for the use of extracts of target substances in the sample. Methanol and formic acid were suitable for mobile phases, and the target compounds could be separated by gradient elution. The method is convenient, rapid and accurate, and can be used for the determination of pyridoxal and pyridoxine in ginkgo seeds. Differences in seed origin and maturity might account for the differences in the contents of pyridoxal and pyridoxine in the selected samples.

## Figures and Tables

**Table 1:** Linear equation, limit of detection and limit of quantification of pyridoxal and pyridoxine.

Compounds	Linear Range (mg/kg)	Linear Equation	R^2^	LODs (mg/kg)	LOQs (mg/kg)
Pyridoxine	0.03–10	Y = 2.1088x − 0.01976	0.9999	0.0065	0.022
Pyridoxal	0.03–10	Y = 1.7995x − 0.01266	0.9999	0.0057	0.019

**Table 2:** Recovery and Precision of the method (n = 6).

Compounds	Background Value (mg/kg)	Spiking Level (mg/kg)	Recovery (%)	RSD (%)
Pyridoxine	ND *	0.6	92.3	1.3
		1.2	91.0	3.4
		6.0	110.4	1.1
Pyridoxal	ND *	0.6	88.8	7.5
		1.2	87.5	5.0
		6.0	86.2	2.8

* ND means Not detected.

**Table 3:** Contents of pyridoxal and pyridoxine in ginkgo seeds from different regions (n = 3).

Origin	Contents	
	Pyridoxal (mg/kg)	Pyridoxine (mg/kg)
Tengchong, Yunnan	0.86 ± 0.02	16.41 ± 0.35
Chengdu, Sichuan	0.56 ± 0.02	0.56 ± 0.09
Pingtian, Guangdong	0.45 ± 0.02	1.38 ± 0.14
Lin’an, Zhejiang	0.74 ± 0.01	1.10 ± 0.02
Guilin, Guangxi	0.82 ± 0.02	0.77 ± 0.03
Taizhou, Jiangsu	0.39 ± 0.03	0.85 ± 0.07
Zunyi, Guizhou	1.26 ± 0.03	26.96 ± 0.40
Linyi, Shandong	0.72 ± 0.05	1.17 ± 0.22
Longnan, Gansu	0.75 ± 0.04	3.31 ± 0.94
Xinyang, Henan	ND *	ND *

* ND means the level below LODs.

## Data Availability

The datasets generated during and/or analyzed during the current study are available from the corresponding author on reasonable request.
